# The CpG Island-Binding Protein SAMD1 Contributes to an Unfavorable Gene Signature in HepG2 Hepatocellular Carcinoma Cells

**DOI:** 10.3390/biology11040557

**Published:** 2022-04-06

**Authors:** Clara Simon, Bastian Stielow, Andrea Nist, Iris Rohner, Lisa Marie Weber, Merle Geller, Sabrina Fischer, Thorsten Stiewe, Robert Liefke

**Affiliations:** 1 Institute of Molecular Biology and Tumor Research (IMT), Faculty of Medicine, Philipps University of Marburg, 35043 Marburg, Germany; clara.simon@imt.uni-marburg.de (C.S.); stielow@imt.uni-marburg.de (B.S.); rohner@staff.uni-marburg.de (I.R.); lisa.weber@imt.uni-marburg.de (L.M.W.); geller@students.uni-marburg.de (M.G.); fische4t@staff.uni-marburg.de (S.F.); 2 Genomics Core Facility, Faculty of Medicine, Institute of Molecular Oncology, Member of the German Center for Lung Research (DZL), Philipps University of Marburg, 35043 Marburg, Germany; andrea.nist@imt.uni-marburg.de (A.N.); stiewe@uni-marburg.de (T.S.); 3 Department of Hematology, Oncology, and Immunology, University Hospital Giessen and Marburg, 35043 Marburg, Germany

**Keywords:** hepatocellular carcinoma, CpG islands, chromatin, SAMD1, transcription, MYC, mTOR, interferon

## Abstract

**Simple Summary:**

Hepatocellular carcinoma (HCC) belongs to the most common cancer types and is the third leading cause of cancer-related deaths. To gain insight into the molecular mechanisms of liver cancer cells, we assessed the role of the CpG island regulator SAMD1, which is highly expressed in liver cancer tissues and associated with poor prognosis. We demonstrate that the deletion of SAMD1 in HepG2 cells leads to aberrant gene regulation and to a gene signature linked to a better prognosis. These results establish SAMD1 as a potentially important player in HCC.

**Abstract:**

The unmethylated CpG island-binding protein SAMD1 is upregulated in many human cancer types, but its cancer-related role has not yet been investigated. Here, we used the hepatocellular carcinoma cell line HepG2 as a cancer model and investigated the cellular and transcriptional roles of SAMD1 using ChIP-Seq and RNA-Seq. SAMD1 targets several thousand gene promoters, where it acts predominantly as a transcriptional repressor. HepG2 cells with SAMD1 deletion showed slightly reduced proliferation, but strongly impaired clonogenicity. This phenotype was accompanied by the decreased expression of pro-proliferative genes, including MYC target genes. Consistently, we observed a decrease in the active H3K4me2 histone mark at most promoters, irrespective of SAMD1 binding. Conversely, we noticed an increase in interferon response pathways and a gain of H3K4me2 at a subset of enhancers that were enriched for IFN-stimulated response elements (ISREs). We identified key transcription factor genes, such as *IRF1*, *STAT2*, and *FOSL2*, that were directly repressed by SAMD1. Moreover, SAMD1 deletion also led to the derepression of the PI3K-inhibitor *PIK3IP1*, contributing to diminished mTOR signaling and ribosome biogenesis pathways. Our work suggests that SAMD1 is involved in establishing a pro-proliferative setting in hepatocellular carcinoma cells. Inhibiting SAMD1’s function in liver cancer cells may therefore lead to a more favorable gene signature.

## 1. Introduction

Hepatocellular carcinoma (HCC) globally belongs to the most common cancer types, is associated with major health-related impairments, and is the third leading cause of cancer-related deaths [1]. Work in recent years has identified numerous molecular targets and signaling pathways that are suitable for treating liver cancer patients [2]. Nonetheless, highly effective antitumor agents are still missing, demonstrating the need to explore further factors as potential therapeutic targets and pharmaceutical treatment options for HCC.

In addition to the aberrant alteration of many signaling pathways [3,4], HCC is also characterized by significant changes in the transcriptional network [5]. In particular, the MYC oncoprotein is often highly expressed in liver cancer cells, which induces aberrant proliferation [6]. MYC affects many different biological processes, such as transcription, translation, and DNA replication [7]. However, despite the important role of MYC, it is considered undruggable [8]. Thus, the identification of molecules and processes that are required for the functioning of MYC may allow the establishment of alternative strategies to target MYC-driven tumors.

We recently identified SAMD1 (SAM domain-containing protein 1) to directly interact with unmethylated CpG islands (CGIs) [9,10], which are key regulatory elements at most gene promoters [11]. SAMD1 is associated with several chromatin modulators, including the histone demethylase KDM1A (lysine-specific histone demethylase 1A) and the chromatin binding protein L3MBTL3 (lethal(3)malignant brain tumor-like protein 3), and it plays a role in gene regulation [9]. In mouse ES cells, the deletion of SAMD1 leads to the derepression of its target genes and an alteration of multiple biological pathways, implicating a pleiotropic role of SAMD1. Indeed, SAMD1 has been proposed to play a role in atherosclerosis [12], was identified in a CRISPR (Clustered Regularly Interspaced Short Palindromic Repeats) screen to be essential for the growth of K562 cancer cells [13], and via a GWAS study, the nonsynonymous variant E338D of SAMD1 was linked to the immune response after malaria infection [14]. Total knockout of SAMD1 leads to impaired angiogenesis and is embryonic lethal [15]. This suggests a potentially versatile and complex biological function of SAMD1. However, the current literature provides only a very limited picture of SAMD1’s role in a physiological and pathophysiological context, prompting us to further examine this underexplored protein.

In the present study, we aimed to gain the first insights into the role of SAMD1 in human cancer cells, with a focus on the hepatocellular carcinoma cell line HepG2, which is a commonly used liver cancer cell line [16,17] and where SAMD1 is strongly expressed. We addressed the consequences of SAMD1 deletion on the biological properties, gene expression, and chromatin landscape of these cells. We found that SAMD1 deletion slightly decreased the proliferation rate, but significantly impaired the clonogenicity of the cells. This phenotype is associated with decreased expression of MYC target genes. Furthermore, we observed an impaired stem cell-like signature. Via genome-wide ChIP-Seq experiments, we confirmed a chromatin regulatory role of SAMD1 in HepG2 cells and showed that it binds to unmethylated CGIs. Moreover, our work indicates that SAMD1 represses the gene transcription of the PI3K (phosphoinositide-3-kinase) interacting protein PIK3IP1 and the tumor suppressor IRF1 (interferon regulatory factor 1), and likely several other factors, to build a transcriptional network that is associated with a poorer prognosis for patients with HCC. Thus, this work supports that interfering with SAMD1’s function in human liver cancer cells could be a valid treatment option for hepatocellular carcinomas.

## 2. Materials and Methods

### 2.1. Cell Culture

HepG2 cells were cultured with MEM, GlutaMAX™ (Thermo Fisher Scientific, Waltham, MA, USA; 41090036) supplemented with 10% fetal bovine serum (Merck; F7524), 1% penicillin–streptomycin (Thermo Fisher Scientific, Waltham, MA, USA; 15140148), and 1× nonessential amino acids (Thermo Fisher Scientific, Waltham, MA, USA; 11140050).

### 2.2. SAMD1 Knockout

SAMD1 knockout in HepG2 cells was conducted via the Lenti-CRISPR system. Two different single guide RNAs targeting SAMD1 (sg1: AGCGCATCTGCCGGATGGTG; sg2: GAGCATCTCGTACCGCAACG) and a nonspecific control single guide RNA were transfected using Opti-MEM™ (Thermo Fisher Scientific, Waltham, MA, USA; 31985062) and FuGENE^®^ HD Transfection Reagent (Promega, Madison, WI, USA; E2311). Single clones were selected using 2 µg/mL puromycin (Merck, Kenilworth, NJ, USA; 58-58-2). The knockout was confirmed by Western blot analysis using a commercial SAMD1 antibody (Bethyl, Montgomery, TX, USA; A303-578A-M).

### 2.3. Nuclear Extract Preparation

To obtain the nuclear extract, the cytoplasmic fraction was removed by incubating harvested cells for 10 min at 4 °C in low salt buffer (10 mM HEPES/KOH pH = 7.9; 10 mM KCl; 1.5 mM MgCl2; 1xPIC (cOmplete™, Protease Inhibitor Cocktail [Roche, Basel, Switzerland; 04693116001]); 0.5 mM PMSF). After centrifugation, the remaining pellet was dissolved in high salt buffer (20 mM HEPES/KOH pH = 7.9; 420 mM NaCl; 1.5 mM MgCl2; 0.2 mM EDTA; 20% glycerol; 1xPIC; 0.5 mM PMSF) and incubated for 20 min at 4 °C while shaking. Subsequently, the lysates were centrifuged, and the supernatant containing the nuclear fraction was further analyzed by Western blotting.

### 2.4. Subcellular Fractionation

A subcellular protein fractionation kit for cultured cells (Thermo Fisher Scientific, Waltham, MA, USA; 78840) was used for fractionation experiments according to the manufacturer’s instructions. A 10 cm dish format was applied, which corresponded to a packed cell volume of 20 µL per well. Localization of SAMD1 was determined using a homemade SAMD1 antibody recognizing the SAM domain [9]. As loading controls for the respective fractions, a homemade SP1 antibody [18], anti-tubulin (Merck, Kenilworth, NJ, USA; MAB3408), and anti-H2Aub (Cell Signaling Technology, Danvers, MA, USA; 8240) were applied. For the detection of histone marks, the chromatin-bound fraction was used. H3 (Abcam, Cambridge, UK; ab1791), H3K4me2 (Diagenode, Denville, NJ, USA; C15410035), H3K4me3 (Diagenode, Denville, NJ, USA; C15410003) and H3K14ac (Abcam, Cambridge, UK; ab52946) antibodies were applied. The signal was quantified using the ImageLab software (v5.2.1, Bio-Rad, Hercules, CA, USA) and normalized to the H3 signal.

### 2.5. Immunofluorescence Staining

For immunofluorescence staining, HepG2 cells were seeded on gelatin-coated coverslips. Cells were fixed with 4% formaldehyde (*w*/*v*), methanol-free (Thermo Fisher Scientific, Waltham, MA, USA; PI28906), and subsequently permeabilized with 0.5% Triton X-100 in PBS. Blocking was performed with 10% FBS in PBS. To detect SAMD1, a homemade SAMD1 antibody recognizing the SAM domain was diluted 1:500 in blocking solution. After primary antibody incubation for 1 h in a wet chamber, the cells were washed three times with 0.5% Triton X-100 in PBS. Secondary antibody incubation was conducted using Alexa Fluor 488 and goat anti-rabbit IgG (H+L) (Thermo Fisher Scientific, Waltham, MA, USA; A-11008) at a 1:1000 dilution. Following three washing steps, the coverslips were mounted onto microscopy slides using VECTASHIELD^®^ Antifade Mounting Medium with DAPI (VECTOR Laboratories, Burlingame, CA, USA; H-1200), and the edges were sealed with nail polish.

### 2.6. Proliferation Assay

To determine the proliferation rates, cells were seeded on 6-well plates at a density of 1 × 10^5^ cells per well. Cell viability was determined 1, 3, and 7 days after seeding using the MTT assay by adding 90 µL of 5 mg/mL thiazolyl blue ≥ 98% (Carl Roth, Karlsruhe, Germany; 4022) to each well. After 1 h, the medium was aspirated, and stained cells were dissolved in 400 µL of lysis buffer (80% isopropanol; 10% 1 M HCl; 10% Triton X-100) and diluted further if necessary. Absorption was measured at 595 nm using a plate reader. All values were normalized to day 1 to compensate for variations in seeding density. The mean value of three biological replicates was determined.

### 2.7. Colony Formation Assay

To examine the ability of cells to form colonies, the cells were seeded at low density (1 × 10^3^ cells per well on 6-well plates) and cultured for 11 days. Next, the cells were washed once with PBS and then fixed with 100% methanol for 20 min. Afterward, the cells were stained for 5 min with 0.5% crystal violet in 25% methanol. To remove excess color, the plates were washed with dH2O until single colonies were visible. Images were taken, and colonies were counted using ImageJ Fiji (v1.53p). The mean value of three biological replicates was determined.

### 2.8. Starvation Assay

To investigate the sensitivity of cells to starvation conditions, cells were seeded on 6-well plates at a density of 1 × 10^5^ cells per well and cultured for 24 h. Next, the culture medium was replaced by medium lacking FBS. A control with standard culture medium was included. After 72 h, cell viability was determined using the MTT assay (see Proliferation Assay).

### 2.9. RNA Preparation

For RNA isolation, cells were cultivated on 6-well plates up to 80–100% confluency. RNA was prepared according to the manufacturer’s manual using the RNeasy Mini Kit (Qiagen, Hilden, Germany; 74004) including an on-column DNA digest.

### 2.10. cDNA Synthesis

The Tetro cDNA Synthesis Kit (Bioline, London, UK; BIO-65043) was used to transcribe mRNA into cDNA according to the manufacturer’s manual. Samples were incubated at 45 °C for 50 min followed by 5 min at 85 °C to inactivate Tetro RT. Subsequently, cDNA was diluted 1:20 to be used in RT-qPCR.

### 2.11. RT-qPCR

For analysis by real-time quantitative PCR, MyTaq™ Mix (Bioline, London, UK; BIO-25041) was used. For gene expression analysis, values were normalized to GAPDH and B2M expression. The qPCR primers used are presented in Supplementary Table S1.

### 2.12. Chromatin Preparation

To prepare chromatin, cells were seeded on 15 cm plates at 3 × 10^6^ cells per plate and cultivated until reaching 70–90% confluence. First, 1% formaldehyde was added to the medium, and the plates were slowly swayed for 10 min to fix the cells. The fixation was stopped by adding 125 mM glycine for 5 min. Subsequently, the cells were washed twice with PBS and scraped in 1 mL cold buffer B (10 mM HEPES/KOH, pH = 6.5; 10 mM EDTA; 0.5 mM EGTA; 0.25% Triton X-100) per 15 cm plate. All plates containing the same cell line were pooled in a 15 mL tube. The tubes were centrifuged for 5 min at 2000 rpm and 4 °C. The supernatant was removed, and the pellet was resuspended in 1 mL cold buffer C (10 mM HEPES/KOH, pH = 6.5 I 10 mM EDTA; 0.5 mM EGTA; 200 mM NaCl) per 15 cm plate followed by a 15 min incubation time on ice. Then, the tubes were centrifuged with the same settings as mentioned before. After removing the supernatant, the pellet was resuspended in 200 µL cold buffer D (50 mM Tris/HCl, pH = 8.0; 10 mM EDTA; 1% SDS; 1xPIC (cOmplete™, Protease Inhibitor Cocktail [Roche, Basel, Switzerland; 04693116001])) per 15 cm plate, vortexed, and incubated for 10–20 min on ice. To shear the chromatin, the samples were sonicated two times for 7 min each using a precooled Bioruptor^®^ (Diagenode, Denville, NJ, USA). The samples were centrifuged for 10 min at 13,000 rpm and 4 °C. The supernatant contained the sheared chromatin.

### 2.13. Chromatin Immunoprecipitation

Chromatin immunoprecipitation (ChIP) for ChIP-qPCR was performed according to the one-day ChIP kit protocol (Diagenode, Denville, NJ, USA; C01010080). For each ChIP, 3 µg of either IgG control antibody (Diagenode, Denville, NJ, USA; C15410206) or homemade SAMD1 antibody recognizing the SAM domain was applied [9]. For the ChIP of histone marks, 1 µg of H3K4me2 antibody (Diagenode, Denville, NJ, USA; C15310035) was used.

To prepare the samples for ChIP sequencing, the one-day ChIP kit protocol was used as described above, but the DNA purification was modified. For DNA elution, the beads were incubated with 230 µL elution buffer (100 mM NaHCO3; 1% SDS) for 30 min at room temperature while shaking. Afterward, the samples were centrifuged at 13,000 rpm for 1 min, and 200 µL of supernatant was transferred to a fresh tube. The input DNA was dissolved in 50 µL of dH2O, and 150 µL of elution buffer was added to obtain an equal volume in all samples. Eight microliters of 5 M NaCl were added to each sample, and the samples were incubated at 65 °C overnight to reverse the cross-linking.

On the next day, 8 µL of 1 M Tris/Cl pH 6.5, 4 µL 0.5 M EDTA, and 2 µL of Proteinase K (10 µg/µL) were added to each sample. All samples were incubated at 45 °C for 1 h while shaking. The DNA was purified using the QIAquick PCR Purification Kit (Qiagen, Hilden, Germany; 28104), whereby all samples prepared with the same antibody were pooled on the same column. To elute the DNA, the columns were incubated for 1 min with 30 µL of sterile 2 mM Tris/Cl, pH 8.5, and centrifuged at 13,000 rpm for 1 min.

The concentration of the samples was determined using the Quant-iT™ dsDNA Assay Kit (Thermo Fisher Scientific, Waltham, MA, USA; Q33120) and the NanoDrop™ 3300 (Thermo Fisher Scientific, Waltham, MA, USA). At least 4 ng of DNA was used for library preparation.

### 2.14. Library Preparation and Next-Generation Sequencing

Next-generation sequencing was performed at the Genomics Core Facility Marburg (Center for Tumor Biology and Immunology, Hans-Meerwein-Str. 3, 35043 Marburg, Germany). For ChIP-Seq, the Microplex library preparation kit v2 (Diagenode, Denville, NJ, USA, C05010012) was used for indexed sequencing library preparation with chromatin immunoprecipitated DNA. Libraries were purified on AMPure magnetic beads (Beckman, Brea, CA, USA; A6388). RNA was prepared as described in “RNA preparation”, and integrity was assessed on an Experion StdSens RNA Chip (Bio-Rad, Hercules, CA, USA; 7007103). RNA-Seq libraries were prepared using the TruSeq Stranded mRNA Library Prep kit (Illumina, San Diego, CA, USA, 2002059). RNA-Seq and ChIP-Seq libraries were quantified on a Bioanalyzer (Agilent Technologies, Santa Clara, CA, USA). Next-generation sequencing was performed on an Illumina NextSeq 550 (Illumina, San Diego, CA, USA).

### 2.15. Bioinformatics Analysis

ChIP-Seq data were aligned to the human genome hg38 using Bowtie [19]. Peak calling was performed with MACS2 with standard settings [20]. The genomic distribution of SAMD1 was determined using ChIPseeker (Galaxy Version 1.28.3) [21]. Gene ontology analysis of SAMD1 target loci was performed using GREAT [22]. Bigwig files, heatmaps, and binding profiles were created using Galaxy/DeepTools [23]. Enhancers were defined as H3K4me2 peaks that did not overlap with promoter sites. Enriched motifs at enhancers were identified using HOMER [24]. ChIP-Seq tracks were visualized using the UCSC browser [25]. Promoter definition and CpG islands were downloaded from the UCSC table browser. The transcription factor list was obtained from http://humantfs.ccbr.utoronto.ca/ (accessed on 11 January 2022) [26].

RNA-Seq samples were aligned to the human transcriptome GENCODE v32 using RNA-Star (2.7.2b) [27]. Reads per gene were calculated using feature counts (2.0.1). Differentially regulated genes and normalized read counts were determined using DESeq2 (2.11.40.6) [28]. Genes with at least 0.5-fold (log2) difference and a *p*-value below 0.01 were considered differentially expressed genes. For the correlation analysis of SAMD1 expression with proliferation, the average expression of 11 proliferation-associated genes (*BIRC5*, *CCNB1*, *CDC20*, *NUF2*, *CEP55*, *NDC80*, *MKI67*, *PTTG1*, *RRM2*, *TYMS*, *UBE2C*) [29] was used. Gene set enrichment analysis (GSEA) [30] was performed with standard settings.

The following internet tools and databases were used: GePIA http://gepia.cancer-pku.cn/ (accessed on 11 January 2022) [22], GePIA2 http://gepia2.cancer-pku.cn/ (accessed on 11 January 2022) [31], ProteinAtlas https://www.proteinatlas.org/ (accessed on 11 January 2022) [32], UALCAN http://ualcan.path.uab.edu/ (accessed on 11 January 2022) [33], HCCDB http://lifeome.net/database/hccdb/home.html (accessed on 11 January 2022) [34], GREAT: http://great.stanford.edu/public/html/ (accessed on 11 January 2022) [35], KMPlotter: https://kmplot.com/ (accessed on 11 January 2022) [36], GALAXY: https://usegalaxy.org/ (accessed on 11 January 2022) [37], and UCSC Browser: https://genome.ucsc.edu/ (accessed on 11 January 2022) [25].

The following public datasets were used: HepG2 WGBS: GSM3633977 [31], HepG2 MYC: GSM822291 [31], HepG2 HES1: GSM803448 [32], HepG2 ATF4: ENCSR669LCD_2, GSE96304 [31], HepG2 H3K4me1: GSM798321 [31], HepG2 H3K4me3: GSM733737 [31], HepG2 RNA Polymerase II: GSM1670896 [33], mESCs MeDIP-Seq: GSM881346 [34], and mESCs ChIP SAMD1: GSM4287311 [9].

### 2.16. Statistical Analysis

The significance of the biological experiments was determined with an unpaired Student’s *t*-test. The significance of gene expression changes was determined by the DESeq2 or GSEA tool. The significance of the gene expression differences between two gene groups was determined via ANOVA. The significance of patient survival was determined by the GePIA tool [22].

## 3. Results

### 3.1. SAMD1 Is Highly Expressed in Liver Cancer and Associated with a Poor Prognosis

**Figure 1:**
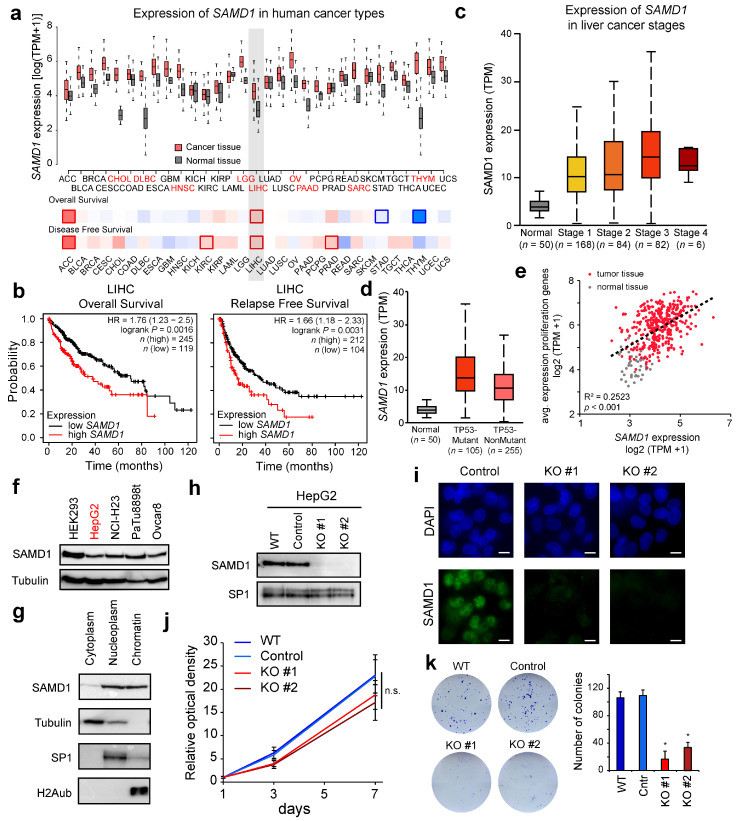
*SAMD1* is highly expressed in liver cancer and is associated with poor prognosis, and its deletion impairs the cellular properties of HepG2 cells. (**a**) Comparison of SAMD1 expression in normal and cancer tissue using data from TCGA, visualized by GePIA [35]. Red cancer names indicate significant upregulation. The lower boxes indicate the hazard ratio of patients’ overall and disease-free survival. Red = high SAMD1 expression correlated with poor prognosis. Blue = high SAMD1 expression correlated with good prognosis. Bold frames indicate significance. (**b**) Kaplan–Meier survival curves show the correlation of SAMD1 expression with patient survival. Plots were made using KM plotter [37]. (**c**) Expression of SAMD1 in distinct liver cancer stages derived from UALCAN [38]. Whisker plots represent the upper and lower quartiles with 5 and 95% whiskers. (**d**) SAMD1 expression in patient samples with and without TP53 mutations compared to healthy liver tissue (“Normal”). Data derived from UALCAN [38]. Box plots represent upper and lower quartiles of the data with 5 and 95% whiskers. (**e**) Correlation of SAMD1 expression with the average expression of 11 proliferation-associated genes in liver tissues. The *p*-value was calculated using ANOVA. (**f**) Western blot of SAMD1 in various human cell lines. HEK293—human embryonic kidney, HepG2—liver cancer, NCI-H23—lung cancer, PaTu8988t—pancreatic cancer, Ovcar8—ovarian carcinoma. The used cell line in this study (HepG2) is marked red. (**g**) Cellular fractionation of HepG2 cells followed by Western blotting. (**h**) Western blot of two SAMD1 knockout clones. (**i**) SAMD1 immunofluorescence in control and SAMD1 KO cells. Bar = 10 µM. (**j**) Proliferation of wild-type, control, and SAMD1 KO HepG2 cells. Error bars indicate the SD of three biological replicates. (**k**) Representative picture of colony formation of wild-type, control, and SAMD1 KO HepG2 cells and quantification. Error bars indicate the SD of three biological replicates. * *p* < 0.05 (Student’s *t*-test). In (**h**–**k**) “#” refers to two distinct SAMD1 KO clones.

We previously characterized SAMD1 as a novel unmethylated CGI-binding protein in mouse ES cells [9], but its function in other biological contexts remained unclear. The investigation of public cancer gene expression data from TCGA showed that the *SAMD1* transcript is mostly upregulated in cancerous tissues compared to normal tissues (Figure 1a) [35]. Significant upregulation of *SAMD1* occurs in cholangiocarcinoma (CHOL), diffuse large B-cell lymphoma (DLBC), head and neck squamous cell carcinoma (HNSC), brain lower-grade glioma (LGG), liver hepatocellular carcinoma (LIHC), ovarian serous cystadenocarcinoma (OV), pancreatic adenocarcinoma (PAAD), sarcoma (SARC), and thymoma (THYM). One of the strongest increases in SAMD1 expression in tumor versus normal tissues was found in liver cancer (Figure 1a). This increased expression can also be observed in other HCC datasets (Supplementary Figure S1a) [36]. The data from TCGA further suggest that high *SAMD1* expression correlates with poor patient survival (Figure 1b) [37]. This association was independent of sex, alcohol consumption, or virus infection (Supplementary Figure S1b). Furthermore, we found that the expression level of *SAMD1* increases in advanced cancer stages and that *SAMD1* expression is highest in cancer samples that also bear a p53 mutation, which is a common liver cancer driver (Figure 1c,d) [38]. The expression of *SAMD1* positively correlates with the expression of common proliferation markers (Figure 1e), suggesting that high SAMD1 levels are associated with higher aggressiveness of cancer.

Thus, these data support the idea that SAMD1 may have a pro-proliferative role in liver cancer cells, which could contribute to worse survival of the patients. Since the role of SAMD1 in cancer has not yet been explored, we decided to investigate the cellular function of SAMD1 in liver cancer cells in greater detail.

### 3.2. SAMD1 Deletion Impairs the Proliferation and Biological Properties of Liver Cancer Cells

Both public data (Supplementary Figure S1c) and Western blotting experiments (Figure 1f) suggest that the SAMD1 protein is abundantly expressed in most human cancer cell lines. To address the role of SAMD1 in liver cancer, we used the commonly applied HepG2 cell line [16], in which SAMD1 can easily be detected via Western blotting (Figure 1f). Notably, contradictory findings were reported about the cellular localization of SAMD1 [10]. In a previous publication, SAMD1 was described to be secreted [12], while our work in mouse ES cells suggested the presence of SAMD1 in the nucleus and bound to chromatin [9]. In HepG2 cells, we also found SAMD1 predominantly in the nucleoplasm and the chromatin fraction, while only a small part was present in the cytoplasmic fraction (Figure 1g). This finding suggests that SAMD1 plays a role in chromatin, not only in mouse stem cells but also in human cancer cells.

To address the potential biological role of SAMD1, we created SAMD1 knockout cells using CRISPR/Cas9 targeting with two different single guide RNAs (sgRNAs), leading to two different KO clones (KO#1 and KO#2). The successful knockout was validated by Western blot and immunofluorescence analysis (Figure 1h,i). SAMD1 knockout HepG2 cells showed a slightly reduced proliferation rate (Figure 1j), consistent with the hypothesis that SAMD1 contributes to liver cancer cell proliferation. To further address the consequence of SAMD1 deletion, we performed colony formation assays, which assessed the ability of the cells to proliferate from single cells. The ability of SAMD1 KO cells to form colonies was significantly reduced in comparison to that of WT and control cells (Figure 1k), suggesting that the absence of SAMD1 strongly impairs the clonogenic survival of HepG2 cells. Presumably, the SAMD1 KO cells require certain factors secreted by other cells for growth, which might explain the different impact of SAMD1 KO on proliferation (Figure 1j) and colony formation (Figure 1k).

Together, these experiments suggest a pivotal role of SAMD1 in maintaining the optimal growth of HepG2 cells.

### 3.3. SAMD1 Deletion Diminishes MYC Target Gene Expression and Stem Cell Signature in HepG2 Cells

**Figure 2:**
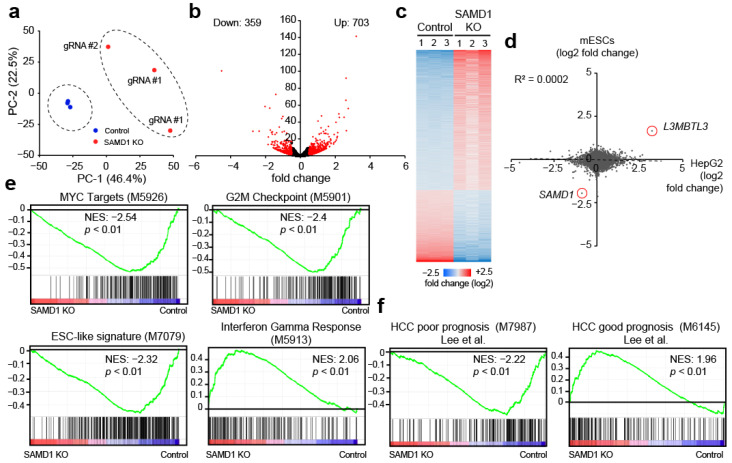
SAMD1 deletion leads to a more favorable transcriptional network. (**a**) Principal component analysis (PCA) of RNA-Seq data from three control samples and three independent SAMD1 KO clones (obtained with two distinct gRNAs, marked by #). (**b**) Volcano plot of RNA-Seq data. Red dots represent significantly differentially expressed genes (*p* < 0.01) with a fold change larger than 0.5 (log2). (**c**) Heatmap of differentially expressed genes from (**b**). (**d**) Comparison of gene expression changes upon SAMD1 deletion in HepG2 cells and mouse ES cells. The scale depicts log2 fold change. (**e**) Unbiased gene set enrichment analysis (GSEA) of RNA-Seq data from HepG2 cells. (**f**) GSEA of a predefined gene set associated with poor or good prognosis in HCC [42].

To understand the reasons why SAMD1 deletion reduces cell proliferation and interferes with cellular properties, we investigated the consequences on gene expression using unbiased RNA-Seq experiments. We used three independent SAMD1 KO clones to avoid clone-specific effects. PCA (principal component analysis) demonstrated that the SAMD1 knockout cells were highly distinct from the control cells (Figure 2a). We identified 762 significantly upregulated and 359 significantly downregulated genes (*p* < 0.01, log2-fold change > 0.5) in the absence of SAMD1 (Figure 2b,c). Interestingly, the most upregulated gene was the *L3MBTL3* gene, which we have already observed in mouse ES cells to become strongly upregulated upon SAMD1 deletion [9]. The L3MBTL3 protein is a direct interacting partner of SAMD1 [9] and is involved in chromatin regulation and transcriptional repression [39,40]. Considering the observation that *L3MBTL3* gene expression is strongly affected in these two unrelated cell lines, we postulate that *L3MBTL3* upregulation could be a compensatory effect and is possibly a common consequence after SAMD1 deletion. We compared the gene expression changes upon SAMD1 deletion in HepG2 and mouse ES cells to address in further detail to what extent the transcriptional consequences upon SAMD1 deletion are similar in these distinct cell lines. We found more pronounced effects in HepG2 cells overall, but surprisingly little correlation between these two cell lines (Figure 2d), in addition to the upregulation of *L3MBTL3* and the downregulation of *SAMD1* itself. These findings suggest that the consequence of SAMD1 deletion is possibly highly cell type-specific.

To investigate the role of SAMD1 in the context of HepG2 cells, we performed unbiased gene set enrichment analyses (GSEA). Initially focusing on common hallmarks, we observed a strong downregulation of MYC target genes (Figure 2e), which are of fundamental importance for cell growth [6] and the maintenance of stem cell characteristics in cancer cells [41]. Consistently, we also found reduced expression of genes related to G2/M checkpoints and ESC-like signatures (Figure 2e, upper right/lower left panel). On the other hand, genes related to the interferon signaling pathway were upregulated (Figure 2e, lower right panel), suggesting multiple opposing consequences upon SAMD1 deletion.

We then asked how the change in gene expression upon SAMD1 deletion may correlate with patient survival. Using previously curated datasets of liver cancer patients [42], we found that gene sets expressed in hepatocellular carcinoma patients with poorer prognosis become predominantly downregulated in SAMD1 KO HepG2 cells, while genes that are expressed in HCC patients with better survival become upregulated (Figure 2f).

Taken together, these data suggest that high SAMD1 expression in liver cancer cells is involved in establishing an unfavorable transcriptional network that contributes to worse prognosis for HCC patients.

### 3.4. SAMD1 Targets Active and Unmethylated CGIs in HepG2 Cells

**Figure 3:**
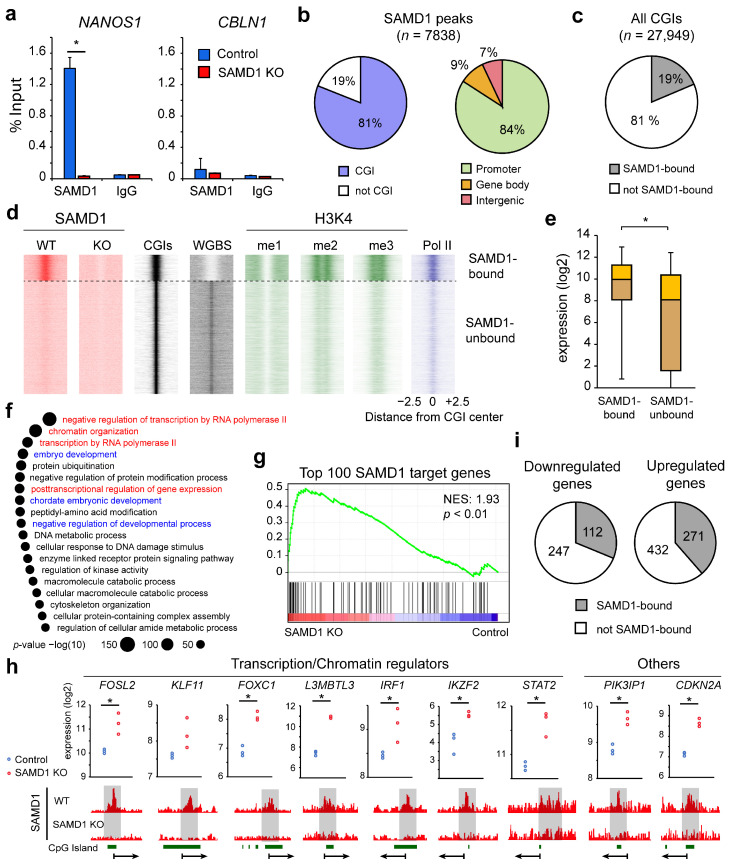
SAMD1 targets unmethylated CGIs in HepG2 cells, where it represses key transcription and signaling factors. (**a**) SAMD1 ChIP-qPCR of the *NANOS1* and *CBLN1* genes in HepG2 cells. Error bars indicate the SD of two technical replicates * *p* < 0.05 (Student’s *t*-test). (**b**) Distribution of significant SAMD1 peaks at CpG islands and promoters, gene bodies, and intergenic regions. (**c**) Distribution of CGIs with and without bound SAMD1. (**d**) Heatmap of all CGIs clustered based on SAMD1 binding. A comparison with DNA methylation (WGBS = whole-genome bisulfite sequencing), active histone marks, and RNA polymerase II (Pol II) is shown [31]. (**e**) Expression of SAMD1-bound and SAMD1-unbound genes. Box plots represent upper and lower quartiles of the data with 5 and 95% whiskers. * *p* < 0.05. (**f**) Gene ontology of the SAMD1-bound genes using GREAT. Transcription-related ontologies are marked in red. Development-associated ontologies are marked in blue. (**g**) GSEA of the top 100 SAMD1 target genes. (**h**) Examples of direct SAMD1 target genes that become upregulated upon SAMD1 deletion. * *p* < 0.01, determined by DeSeq2. (**i**) Distribution of SAMD1-bound genes within the up- and downregulated genes.

Our previous work in mouse ES cells suggested that SAMD1 functions as a transcriptional repressor at actively transcribed and unmethylated CGIs [9]. To assess whether SAMD1 also binds to CGI-containing promoters in HepG2 cells, we performed ChIP-qPCR experiments and confirmed that SAMD1 binds to the *NANOS1* gene, which is one of its top targets in mouse ES cells (Figure 3a) [9]. In contrast, we did not find SAMD1 binding at *CBLN1*, another top target in mouse ES cells (Figure 3a). This inconsistent binding behavior implicates a dissimilar binding pattern of SAMD1 in HepG2 and mouse ES cells.

To obtain a better picture of SAMD1 chromatin binding in HepG2 cells, we elucidated the genomic targets of SAMD1 via ChIP-Seq. Comparing the control and SAMD1 knockout cells, we identified 7838 regions with significantly enriched SAMD1 recruitment. Consistent with our results in mouse ES cells, SAMD1 was almost exclusively present at CpG islands and consequently at promoters in HepG2 cells (Figure 3b). Given that only a subset of CpG islands is targeted by SAMD1 (Figure 3c), we investigated the properties of SAMD1-bound CGIs in greater detail. Using public HepG2 DNA methylation data from the ENCODE consortium [31], we found that SAMD1-bound CGIs are mostly unmethylated (Figure 3d), consistent with our finding that SAMD1 is repelled by methylated DNA [9]. Further analysis showed that SAMD1-bound CGIs are decorated with active histone marks and show high levels of RNA polymerase II (Figure 3d), indicating that SAMD1 binds to transcriptionally active CGIs. Consistently, we found that the corresponding genes were highly expressed (Figure 3e). Gene ontology analysis using GREAT demonstrated that the SAMD1-targeted loci are involved in multiple cellular processes, including transcriptional regulation, chromatin organization, and developmental processes (Figure 3f). Thus, SAMD1 may play a pivotal role in establishing a specific chromatin landscape in HepG2 cells by modulating the expression of the highly active genes that contribute to chromatin organization.

Next, we addressed to what extent cell type-specific characteristics contribute to the chromatin binding of SAMD1. For this purpose, we compared the binding pattern of SAMD1 in HepG2 cells with that from our previous study in mouse ES cells [9]. SAMD1 levels were calculated for all gene promoters that existed in both organisms (Supplementary Figure S2a). We identified approximately 600 promoters, such as the *Nanos1*/*NANOS1* promoter, which are highly bound by SAMD1 in both cell types, but we also identified approximately 1000 promoters that are only bound by SAMD1 in one of the two cell lines (Supplementary Figure S2a). For example, the *Cbln1/CBLN1* promoter, which is the top SAMD1 target in mouse ES cells [9], was not bound in HepG2 cells (Supplementary Figure S2b), confirming our results from the ChIP-qPCR experiment (Figure 3a). In contrast, the promoters of *Prnp/PRNP* and *Ankrd36/ANKRD36* were SAMD1-bound in HepG2 cells, but not in mouse ES cells (Supplementary Figure S2b). This finding suggests that SAMD1 binding differs depending on the biological and genomic context. To address this in more detail, we investigated the DNA methylation levels and the presence of CGIs at each promoter (Supplementary Figure S2c,d). We found that genes that are bound in mouse ES cells, but not in HepG2 cells, differ mainly regarding their DNA methylation status. These genes are unmethylated in mouse ES cells but are mostly methylated in HepG2 cells. Conversely, genes that are bound in HepG2 cells but not in mouse ES cells are mostly unmethylated in both cell types but differ strongly regarding the CGI content at the promoters. They often have large CGIs in humans, but smaller or no CGIs in mice. The *Prnp*/*PRNP* and *Ankrd36*/*ANKRD36* promoters are examples of this phenomenon (Supplementary Figure S2a,b). Together, these results suggest that SAMD1 binds most efficiently to promoter CGIs that are large and unmethylated. Thus, dependent on the methylation status and CGI sizes, it is likely that the SAMD1 binding pattern differs from organism to organism and cell type to cell type. These differences may partially explain why the gene expression changes upon SAMD1 deletion in mouse ES cells and human HepG2 cells showed hardly any correlation (Figure 2d).

### 3.5. SAMD1 Directly Represses Key Transcription Factors and Signaling Regulators

To gain further insights into how SAMD1 impacts gene expression in HepG2 cells, we assessed the transcriptional changes of SAMD1-bound genes. Using our RNA-Seq data, we found that the top 100 SAMD1 target genes were preferentially upregulated upon SAMD1 deletion (Figure 3g), consistent with our previous observation that SAMD1 mainly functions as a transcriptional repressor at highly expressed genes [9]. Thus, SAMD1 may counteract activating regulators at its target genes in HepG2 cells.

Genes that are bound by SAMD1 and become derepressed upon SAMD1 deletion are likely direct downstream targets of SAMD1 in HepG2 cells. Consistent with the gene ontology analysis (Figure 3f), this group of genes includes many transcription factors (*IRF1*, *FOXC1*, *IKZF1*, *FOSL2*, *STAT2*) and chromatin regulators (*L3MBTL3*) (Figure 3h). Furthermore, we identified the *CDKN2A* gene, which encodes the tumor suppressors p16INK4a and p14ARF [43], and *PIK3IP1* as direct targets of SAMD1 (Figure 3h).

Interestingly, only approximately one-third of the genes affected in gene expression after SAMD1 KO in HepG2 cells were bound by SAMD1 itself, and up- or downregulated genes were similarly occupied by SAMD1 (Figure 3i). These findings suggest that many of the observed gene expression changes are not directly related to SAMD1 chromatin binding but are due to indirect effects. Therefore, we hypothesized that the derepression of direct SAMD1 target genes may influence signaling and transcriptional pathways, which, in turn, alter further downstream targets.

### 3.6. SAMD1 Deletion Increases Susceptibility of HepG2 Cells to Serum Starvation

**Figure 4:**
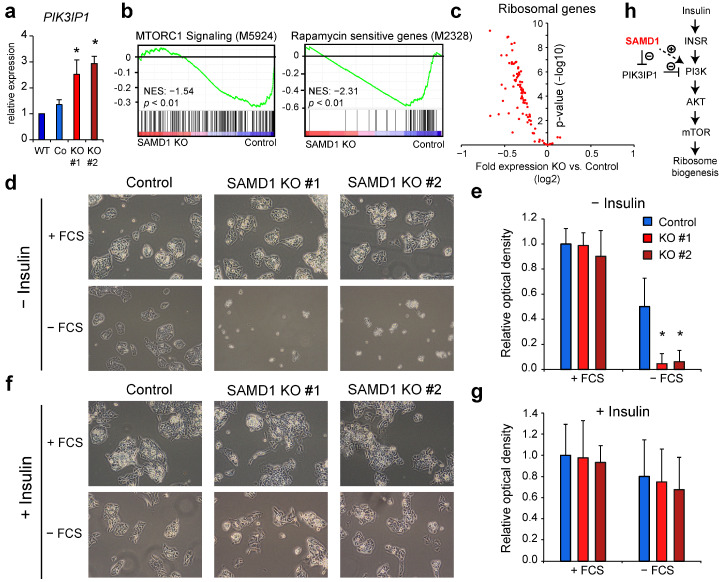
SAMD1 deletion impairs the mTOR signaling pathway and sensitizes cells to serum starvation. (**a**) Expression of *PIK3IP1* in wild-type, control, and SAMD1 KO cells, measured via RT-qPCR. Error bars indicate the SD of three biological replicates. * *p* < 0.05 (Student’s *t*-test) (**b**) GSEA of mTOR signaling and rapamycin-sensitive genes in SAMD1 KO cells. (**c**) Volcano plot of ribosomal genes comparing expression in control and SAMD1 KO cells. (**d**) Representative bright-field microscopy of SAMD1 KO and control HepG2 cells upon serum starvation. (**e**) MTT viability assay of cells treated as in (**d**). Error bars indicate the SD of three biological replicates. * *p* < 0.05 (Student’s *t*-test). (**f**) Representative bright-field microscopy of SAMD1 KO and control HepG2 cells upon serum starvation supplemented with insulin. (**g**) MTT viability assay of cells treated as in (**f**). Error bars indicate the SD of three biological replicates. * *p* < 0.05 (Student’s *t*-test). (**h**) Model of SAMD1’s influence on the mTOR signaling pathway. In (**a**,**d**–**g**) the “#” refers to two distinct SAMD1 KO clones.

As described above, SAMD1 deletion leads to the upregulation of its direct target *PIK3IP1*. We confirmed the results from the RNA-Seq analysis via RT-qPCR (Figure 4a). The PIK3IP1 protein acts as an inhibitor of phosphatidylinositide-3-kinases (PI3Ks) [44], which are key kinases of many pro-proliferative signaling pathways [45] and play a central role in the insulin signaling pathway in liver cells [46]. The inhibition of PI3Ks through PIK3IP1 has been shown to inhibit DNA synthesis and the survival of hepatocytes and to suppress the development of hepatocellular carcinoma [47].

We asked whether the increased expression of *PIK3IP1* upon SAMD1 deletion may impair the mTOR signaling pathway, which is downstream of PI3K and a key regulator of ribosome biogenesis and translation [48]. Increased mTOR signaling often occurs in liver cancer and is involved in drug resistance, which is why it has been proposed to be suitable for drug targeting approaches [49,50]. Gene set enrichment analysis demonstrated that mTORC1 signaling was indeed downregulated in SAMD1 KO cells (Figure 4b). Consistently, we found a strong downregulation of rapamycin-sensitive genes (Figure 4b), and the majority of ribosomal genes showed reduced expression in these cells (Figure 4c), supporting that SAMD1 knockout cells are characterized by impaired mTOR signaling and ribosome biogenesis.

Upon serum starvation, the mTOR signaling pathway is typically inactivated, which leads to autophagy to compensate for the reduced energy supply [51]. We hypothesized that impaired mTOR signaling in SAMD1 KO cells may lead to increased sensitivity to reduced serum levels. Consistent with this idea, we found that SAMD1 KO cells that were starved for 72 h showed significantly decreased cell viability compared to control cells (Figure 4d,e), suggesting that SAMD1 KO cells have a lower capacity to respond to starvation. Importantly, insulin supplementation during starvation almost completely abolished the negative effect of SAMD1 KO (Figure 4f,g). This finding supports the idea that reduced mTOR signaling in SAMD1 KO cells can be compensated by artificially activating the insulin pathway under starvation conditions.

Taken together, these experiments support the model in which SAMD1 acts as a negative regulator of *PIK3IP1* in wild-type HepG2 cells, which is a negative regulator of PI3K in hepatocellular carcinoma cells [47]. Thus, high SAMD1 expression could contribute to an augmentation of the mTOR signaling pathway in liver cancer cells (Figure 4h), which may lead to enhanced cell growth [52].

### 3.7. SAMD1 Deletion Leads to Reduced H3K4me2 Levels at Most Promoters

**Figure 5:**
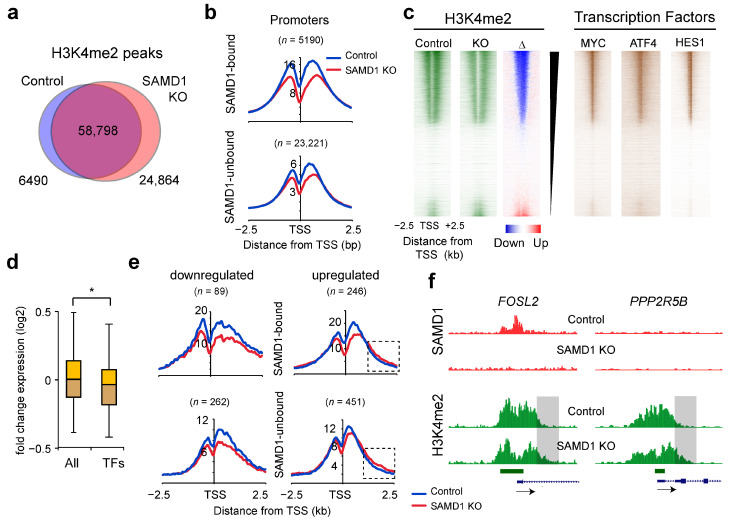
SAMD1 deletion leads to reduced H3K4me2 promoter levels independent of SAMD1 occupancy. (**a**) Venn diagram showing the overlap of significant H3K4me2 peaks in control and SAMD1 knockout cells. (**b**) H3K4me2 ChIP-Seq promoter profiles at SAMD1-bound and SAMD1-unbound promoters. (**c**) Heatmap showing the overlap of reduced H3K4me2 with three example transcription factors. (**d**) Gene expression changes of transcription factors [26] (*n* = 1386) in SAMD1 KO cells compared to all genes. Box plots represent upper and lower quartiles of the data with 5 and 95% whiskers. Significance was evaluated using ANOVA. * *p* < 0.01. (**e**) H3K4me2 ChIP-Seq promoter profiles of up- and downregulated genes. (**f**) Example ChIP-Seq data of upregulated genes that show increased H3K4me2 downstream of the TSS.

Because SAMD1 also represses many transcription factors (Figure 3f,h), we next investigated the impact on the chromatin landscape. For this purpose, we assessed the genome-wide distribution of the active histone mark H3K4me2 in control and SAMD1-deleted cells. The H3K4me2 mark is present both at promoters and enhancers, allowing us to obtain a comprehensive picture of global changes in the chromatin landscape. Using the obtained data, we identified 65,288 significant H3K4me2 peaks in the control cells and 83,662 in the knockout cells (Figure 5a). Of those peaks, 58,798 were shared in both cell lines, while 24,864 peaks were specific for the knockout cells and 6490 for the control cells.

At the promoters, H3K4me2 levels were reduced, independent of SAMD1 binding (Figure 5b). These promoter-specific effects could be an indirect consequence of SAMD1 deletion and are probably not related to its chromatin regulatory role [9]. When classifying the promoters regarding their H3K4me2 changes, we found that the promoters with strongly reduced H3K4me2 are occupied by many transcription factors, such as MYC, HES1, and ATF4, while promoters that have increased H3K4me2 appear less targeted by transcription factors (Figure 5c). Although several transcription factor genes were repressed by SAMD1 (Figure 3h), they were, on average, downregulated in SAMD1 KO cells when all transcription factors were taken into account (Figure 5d). Among them are the genes for the abovementioned transcription factors MYC (−0.39-fold change (log2)), HES1 (−0.81), and ATF4 (−0,46). This suggests that SAMD1 deletion indirectly impairs many transcription factor-dependent processes, leading to reduced H3K4me2 at most promoters. Consistent with this global reduction of H3K4me2 at promoters, we also observed a reduced H3K4me2 level in the SAMD1 knockout cells by Western blotting, while other histone marks such as H3K4me3 and H3K14ac appear to be less affected (Supplementary Figures S3 and S4).

Interestingly, genes that have increased expression after SAMD1 deletion often show increased H3K4me2 levels downstream of the TSS (Figure 5e,f). This effect is independent of SAMD1 binding, as well. Thus, at upregulated genes, other chromatin regulatory mechanisms, such as chromatin remodeling processes, are possibly altered after SAMD1 deletion. This finding suggests that several independent chromatin regulatory processes at promoters are affected after SAMD1 deletion. Given the possibility that many of them are indirectly influenced by SAMD1, this cannot be easily dissected.

### 3.8. SAMD1 Deletion Activates Enhancers Enriched for Interferon-Stimulated Response Elements

**Figure 6:**
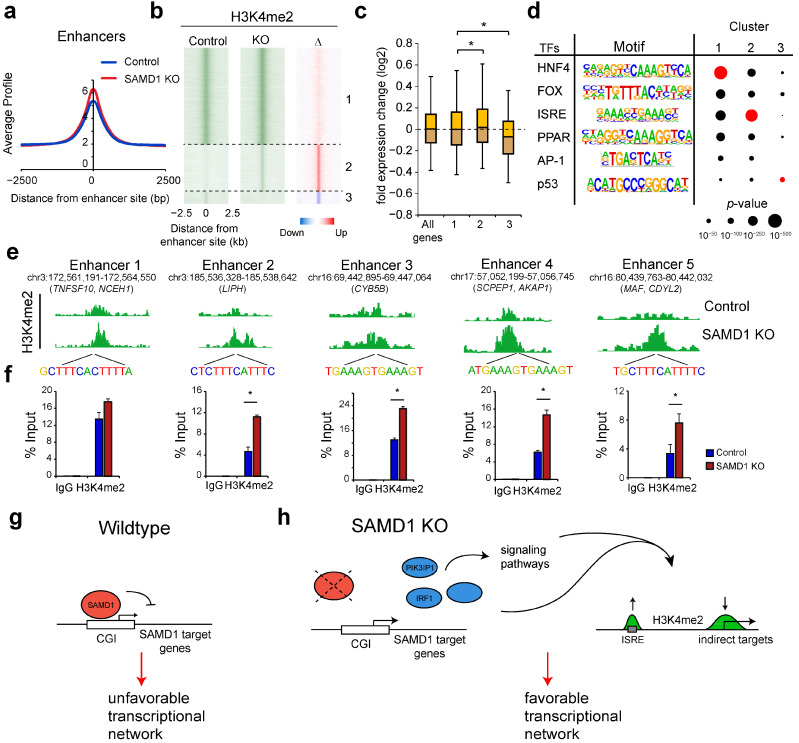
SAMD1 deletion leads to increased H3K4me2 levels on a subset of enhancers. (**a**) H3K4me2 ChIP-Seq profiles in control and SAMD1 KO cells at all enhancers. (**b**) Heatmap of H3K4me2 at enhancers. The difference is shown in the right plot. Three clusters were defined based on levels in control and knockout cells. (group 1: *n* = 49,133; 2: *n* = 23,960; 3: *n* = 6160) (**c**) Gene expression changes of the neighboring genes to the enhancers in each group, compared to all genes. Box plots represent upper and lower quartiles of the data with 5 and 95% whiskers. Significance was evaluated using ANOVA. * *p* < 0.01. (**d**) Motif analysis of the three clusters from B. The motif that is most strongly enriched in each cluster is marked red. (**e**) H3K4me2 ChIP-Seq data at example enhancers with increased H3K4me2 after SAMD1 deletion. (**f**) ChIP-qPCR of H3K4me2 at these enhancers in control and SAMD1 KO cells. Error bars indicate the SD of two technical replicates. * *p* < 0.05 (Student’s *t*-test). (**g**) Model of SAMD1’s role in hepatocellular carcinoma cells. In wild-type HepG2 cells, SAMD1 binds to unmethylated CpG islands functioning as a transcriptional repressor and contributing to an unfavorable transcriptional network. (**h**) Upon SAMD1 deletion, SAMD1 target genes such as *PIK3IP1* and *IRF1* become derepressed, which affects signaling pathways and further downstream targets. This leads to a reduced H3K4me2 level at most promoters and an increase in H3K4me2 levels at enhancers related to the interferon response. Together, this establishes a more favorable transcriptional setup.

At enhancers where SAMD1 is not binding, we also observed substantial changes. Looking at all enhancer sites (*n* = 79,253), we observed, on average, an increased level of H3K4me2 (Figure 6a), opposite to the consequences found at the promoters (Figure 5b). A large group of enhancers is H3K4me2 decorated in both cell lines (group 1, *n* = 49,133). Nevertheless, we also identified many enhancers with low H3K4me2 in the control cells but increased H3K4me2 in the SAMD1 knockout cells (group 2, *n* = 23,960) (Figure 6b). In contrast, we found many fewer enhancers that lost H3K4me2 upon SAMD1 deletion (group 3, *n* = 6160) (Figure 6b). Consistent with the known function of enhancers [53], the alteration of enhancer H3K4me2 levels also correlates with the gene expression changes of nearby genes, supporting that the regulation of the enhancer landscape plays a major role in the observed gene expression changes.

To gain insights into how SAMD1 deletion influences the enhancer landscape, we performed motif enrichment analysis (Figure 6d). At enhancers that are active in both cell lines, we found that the motif for HNF (hepatocyte nuclear factor) was most strongly enriched. This finding is not unexpected given that HNF4 is a major transcriptional regulator of liver cells [54,55]. Additionally, the interferon-stimulated response element (ISRE), as well as the motifs for PPAR and AP-1 transcription factors, were enriched. The enhancers that gain H3K4me2 upon SAMD1 deletion have a similar set of enriched motifs, but instead of the HNF motif, the ISRE motif is most strongly enriched (Figure 6d). We confirmed an increase in H3K4me2 at some of those enhancers via ChIP-qPCR (Figure 6e). The finding that these ISRE-possessing enhancers have increased H3K4me2 in SAMD1 KO cells supports our previous observation that augmented interferon signaling occurs in these cells (Figure 2e). The increased expression of IRF1 and STAT2 (Figure 3h), which bind to this motif [56], may contribute to this phenomenon.

In summary, our RNA-Seq and ChIP-Seq experiments suggest a global readjustment of the transcriptional and chromatin landscape in HepG2 cells after SAMD1 deletion, leading to an altogether more favorable gene signature (Figure 2f).

## 4. Discussion

Our work demonstrated that SAMD1 is commonly upregulated in liver cancer and that its high expression is associated with poor prognosis in this cancer type (Figure 1a,b). Given the detectable expression of SAMD1 in HepG2 liver cancer cells (Figure 1f), these cells were selected as our experimental model for further investigations. The HepG2 cell line is widely used as a liver cancer model and shares common characteristics with patient liver cancers [16,17]. These cells were also included in comprehensive epigenome analyses by the ENCODE project [31], allowing us to compare our data with other genomic features. Using HepG2 cells, we addressed the question of how the upregulation of SAMD1 in liver cancer may contribute to liver cancer progression and poor prognosis.

Upon deletion of SAMD1, we observed a slightly reduced proliferation capacity and strongly impaired colony-forming ability (Figure 1j,k). This observation suggests that the absence of SAMD1 substantially impairs the biological function of these cells, particularly the ability to grow colonies out of one cell. Indeed, the analysis of the gene expression changes upon SAMD1 deletion suggests that several key processes are affected (Figure 2e). Specifically, we observed a downregulation of common cancer-related gene sets, such as MYC target genes and genes involved in an ESC-like signature. In contrast, we observed an increase in interferon response genes. Importantly, the investigation of common signatures that have been associated with good or poor prognosis [42] suggests that SAMD1 deletion alters the transcriptional network towards a signature that would be preferable for liver cancer patients (Figure 2f).

Unexpectedly, the gene expression changes that we observed upon SAMD1 deletion did not correlate well with the changes that we had observed before in mouse ES cells (Figure 2d). In addition to SAMD1 itself, the only gene that was clearly affected in both cell lines was *L3MBTL3*. Given that the L3MBTL3 protein is a direct interaction partner of SAMD1 [9], the deletion of SAMD1 may be compensated by the increased expression of L3MBTL3 via an unknown feedback mechanism. The otherwise low correlation in the gene expression changes suggests that the gene regulatory function of SAMD1 is possibly strongly dependent on the cellular context, and that the results from one cell type cannot be easily transferred to other biological systems.

The genome-wide analysis of the SAMD1 binding pattern provided us with further details on how SAMD1 regulates gene expression in HepG2 cells. Consistent with our biochemical studies indicating that the DNA-binding winged-helix domain of SAMD1 prefers unmethylated CpG motifs [9], SAMD1 exclusively binds to unmethylated CGIs in HepG2 cells. These CGIs are typically highly active, meaning they are decorated by active histone marks, and the associated genes are highly expressed (Figure 3d,e). The investigation of the SAMD1 binding patterns in mouse ES cells and human HepG2 cells further suggests that SAMD1 preferentially binds to large, unmethylated CpG islands (Supplementary Figure S2). The self-association ability of SAMD1 via its SAM domain [9] may allow the cooperative binding of SAMD1 to several unmethylated CpG motifs, which might work best at larger CGIs.

We found that SAMD1-bound genes were preferentially upregulated upon SAMD1 deletion (Figure 3g), supporting the previously observed repressive role of SAMD1 [9]. The repressive function of SAMD1 may include the function of the KDM1A histone demethylase [9], but other mechanisms, such as the function of SAMD1 interacting with the MBT domain-containing proteins L3MBTL3, SFMBT1, and SFMBT2, may also be involved [9,39,57]. Additionally, the potential SAM polymerization ability of SAMD1 could play a role in the function of SAMD1 as a transcriptional repressor at CGIs [9]. Thus, it will be of interest to further decipher the molecular mechanisms of SAMD1’s repressive function in human cancer cells in future studies.

One of the major direct target genes of SAMD1 appears to be the gene *PIK3IP1,* where SAMD1 may cooperate with KDM1A to regulate gene transcription [9,58]. *PIK3IP1* functions as an inhibitor of PI3 kinases [44,47], thus acting upstream of the PI3K/Akt/mTOR pathway. This pathway is commonly dysregulated in HCC and has been a major target for therapeutic interventions in HCC [50,52]. High *PIK3IP1* expression is associated with better prognosis [47], suggesting that *PIK3IP1* upregulation may contribute to the shift of the transcriptional landscape towards a more favorable setting. Our data suggest that impaired mTOR signaling and reduced expression of ribosome biogenesis genes are important features of HepG2 SAMD1 knockout cells.

In addition to regulating signaling pathways, SAMD1 deletion appears to strongly influence the chromatin landscape. For an initial characterization, we focused on the active H3K4me2 histone mark. Surprisingly, we observed opposite effects at promoters and enhancers. We found a decrease in H3K4me2 at promoters, while we mostly observed an increase at enhancers. In both cases, these changes appear to be largely independent of SAMD1 presence, since most changes occur at SAMD1-bound and SAMD1-unbound loci. This observation implies that most of the alterations in the chromatin landscape are due to the indirect modulations of chromatin regulatory mechanisms. Consistently, only approximately one-third of dysregulated genes are bound by SAMD1.

One of these indirect effects may involve the IRF1 protein, which is a direct target of SAMD1 and is upregulated in SAMD1 KO cells (Figure 3h). IRF1 has been described as a tumor suppressor in many cancer types and suppresses MYC-driven oncogenesis [59,60]. The precise mechanism of action of IRF1 has not yet been fully explored, but likely includes both repressive and activating functions [61,62]. IRF1 also plays an important role in the interferon response [63], which may explain the increased expression of genes related to interferon signaling (Figure 2e) and the increased level of H3K4me2 at enhancers with interferon response elements (Figure 6b,d). IRF1 has also been linked to a better prognosis in HCC [64], supporting a relevant role of IRF1 in liver cancer cells.

Another interesting candidate found to be dysregulated was the gene *CDKN2A*, encoding the tumor suppressors p14ARF and p16INK4a. The *CDKN2A* gene is often dysregulated in hepatocellular carcinomas by promoter methylation [65]. In addition to IRF1, PIK3IP1, and CDKN2A, SAMD1 likely affects many other critical factors that further influence downstream pathways (Figure 6h). To gain an understanding of the direct effects of SAMD1 deletion on the chromatin landscape, it will be necessary to track immediate changes upon SAMD1 removal.

Nonetheless, our work suggests that the absence of SAMD1 has a major impact on the transcriptional and chromatin landscape in HepG2 cells. The high expression of SAMD1 in these cells contributes to a pro-proliferative setting that is required for optimal growth. The absence of SAMD1 leads to a more favorable transcriptional signature, which is linked to better prognosis (Figure 2f) [42]. Whether this signature could also improve the response to chemotherapeutic drugs remains to be determined.

In addition to liver cancer, SAMD1 is upregulated in many other human cancer types, suggesting that SAMD1 could play a pivotal role in various cancers (Figure 1a). Notably, given conflicting gene annotations [10], the human SAMD1 gene has not been included in comprehensive CRISPR screening experiments that investigated the role of most human genes in human cancer cell lines [66]. However, in the limited number of CRISPR screens that included SAMD1, it often scores highly [13,67], suggesting a functional role of SAMD1 in multiple biological processes. In the future, it will also be of interest to address whether SAMD1 plays a role in other human cancer cell types and to analyze its involvement during tumor onset.

The work presented here has several limitations. First, all experiments were performed in one cell line: HepG2 cells. Consequently, we cannot exclude the possibility that the observed biological effects and gene expression changes are different in other liver cancer cell lines or in liver cancer tissues. Given that we observed almost no correlation between the gene expression changes in mouse ES cells and HepG2 cells (Figure 2d), it is likely that the role of SAMD1 is highly cell type-specific. Therefore, it is not possible to extrapolate our findings to other cells until further data are collected. Our characterization of the chromatin landscape is restricted to the H3K4me2 mark, thus providing only a limited picture of changes in the chromatin state. It is possible that further chromatin marks, including repressive histone modifications, are globally altered upon SAMD1 removal, which will be of interest to be addressed in future studies. Furthermore, it is currently unclear which of the direct targets of SAMD1 are most relevant for the observed phenotypes. Additionally, we cannot exclude the possibility that some of the observed effects are due to the chromatin-independent roles of SAMD1. Further work is required to fully comprehend SAMD1’s role in liver and other cancer types.

## 5. Conclusions

Collectively, our work demonstrates that SAMD1 modulates the transcription of unmethylated CGI-containing genes in HepG2 cells, which contributes to the establishment of an unfavorable transcriptional network (Figure 6g). This finding may explain why high SAMD1 expression is associated with worse prognosis. Interfering with SAMD1’s function may be suitable to shift liver cancer cells towards a more favorable setup (Figure 6h), which could provide a new strategy for the treatment of liver cancer patients.

## Data Availability

ChIP-Seq and RNA-Seq are openly available at the GEO repository, reference number GSE190761. Other data are available on request from the corresponding author.

## Supplementary Materials

The following supporting information can be downloaded at: https://www.mdpi.com/article/10.3390/biology11040557/s1, Figure S1: Further investigation of SAMD1 in liver cancer; Figure S2: Comparison of SAMD1 chromatin binding in human HepG2 and mouse ES cells; Figure S3: Global levels of histone modifications in SAMD1 KO HepG2 cells; Figure S4: Uncropped original images; Table S1: qPCR Primers.
