# Supplementary material for: The CpG Island-Binding Protein SAMD1 Contributes to an Unfavorable Gene Signature in HepG2 Hepatocellular Carcinoma Cells
Source: Biology (Basel). 2022 Apr 6;11(4):557. doi: 10.3390/biology11040557 (PMC9032685; doi:10.3390/biology11040557)
Supplement: Supplementary file 1 [file biology-11-00557-s001.zip › biology-1624215-SupplementaryData_Final-done.pdf]

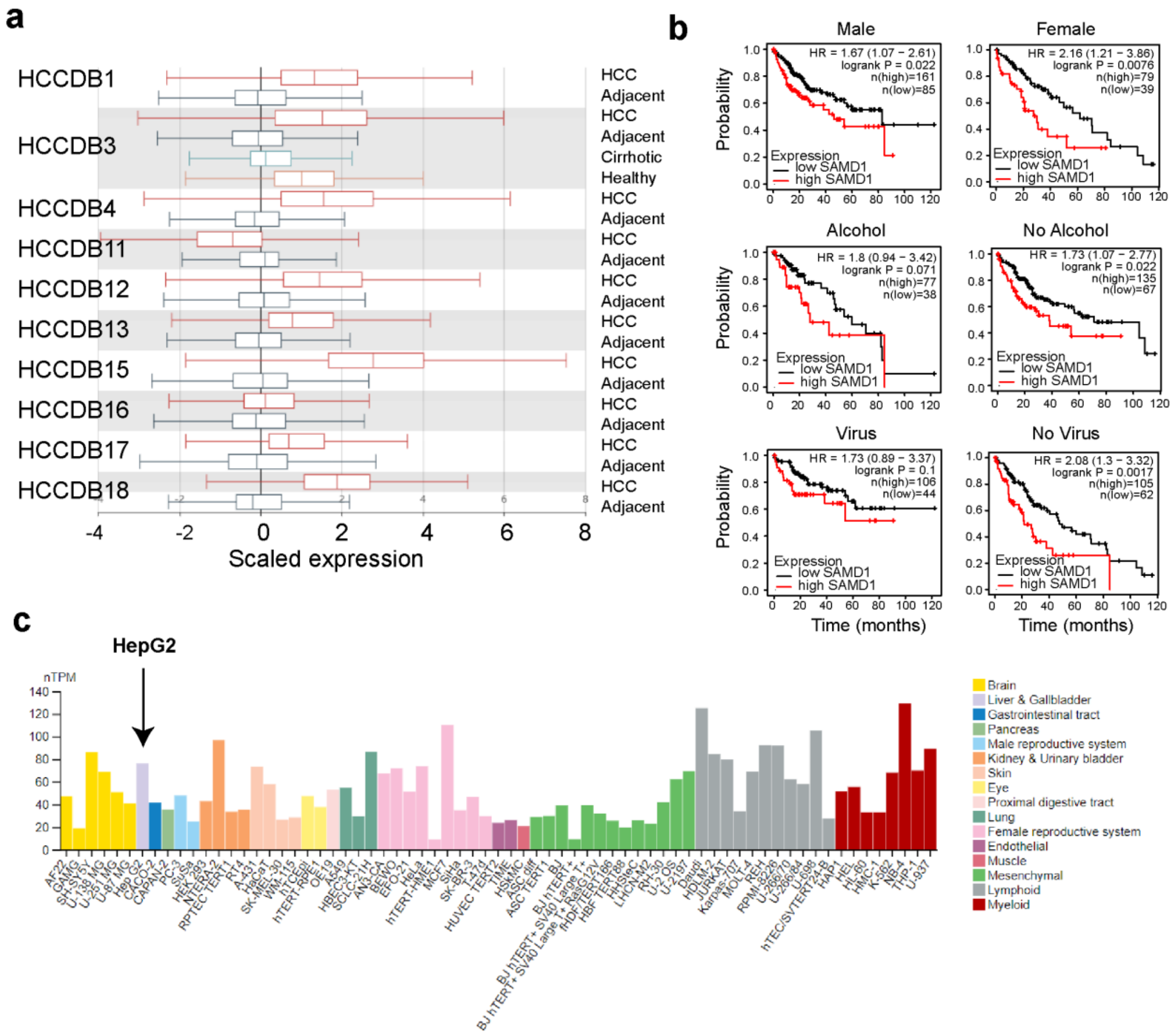

Figure S1. Further investigation of SAMD1 in liver cancer. **a** SAMD1 expression in liver cancer samples compared to healthy tissue in several independent HCC cohorts. Data derived from HCCDB[16]. **b** Patient survival from distinct cohorts with low and high SAMD1 expression. Plots were created using KM plotter[17]. **c** Expression of SAMD1 in human cancer cell lines. Date derived from Proteinatlas[19].

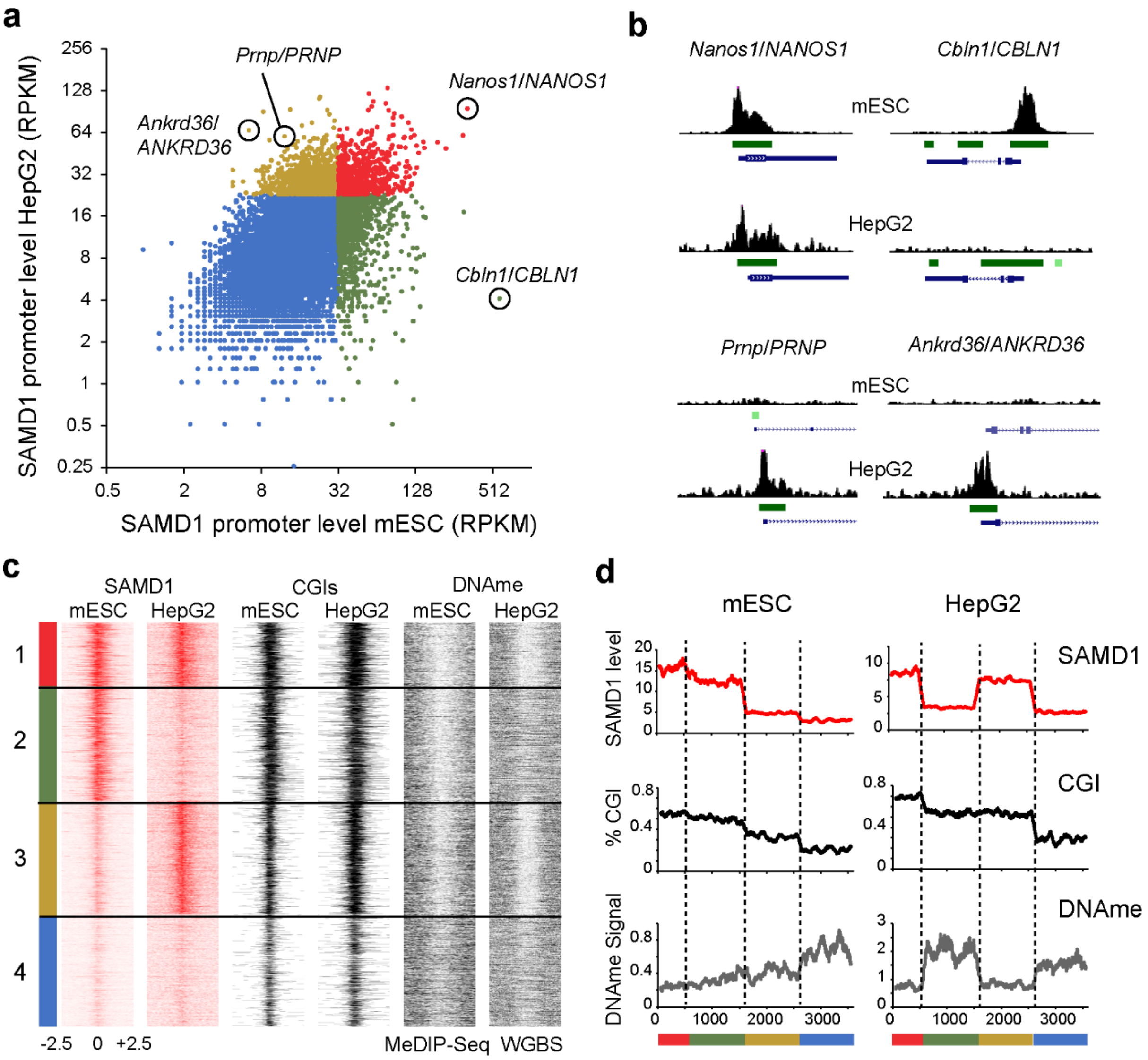

Figure S2. Comparison of SAMD1 chromatin binding in human HepG2 and mouse ES cells. **a** Correlation of SAMD1 promoter levels in mouse ES cells (mESCs) and human HepG2 cells. Only promoters that existed in both organisms were included. Four promoter groups were defined based on their SAMD1 levels in both cell lines. Group 1 (n= 586, red): bound in both cell types. Group 2 (n = 1002, green) bound only in mouse ES cells. Group 3 (n = 1016, yellow): bound only in HepG2 cells. Group 4 (n = 12,946, blue): not strongly bound in either cell type. **b** Examples of promoters that are bound by SAMD1 in both cell types, only in mouse ES cells or only in HepG2 cells. **c** Heatmap of the four groups from A in comparison with the presence of CGIs and DNA methylation. MeDIP-Seq = Methylated DNA immunoprecipitation sequencing. For group 4, 1000 randomly selected promoters were used. **d** Signal strength at each promoter group as in c, presented as a sliding window average (100 promoters).

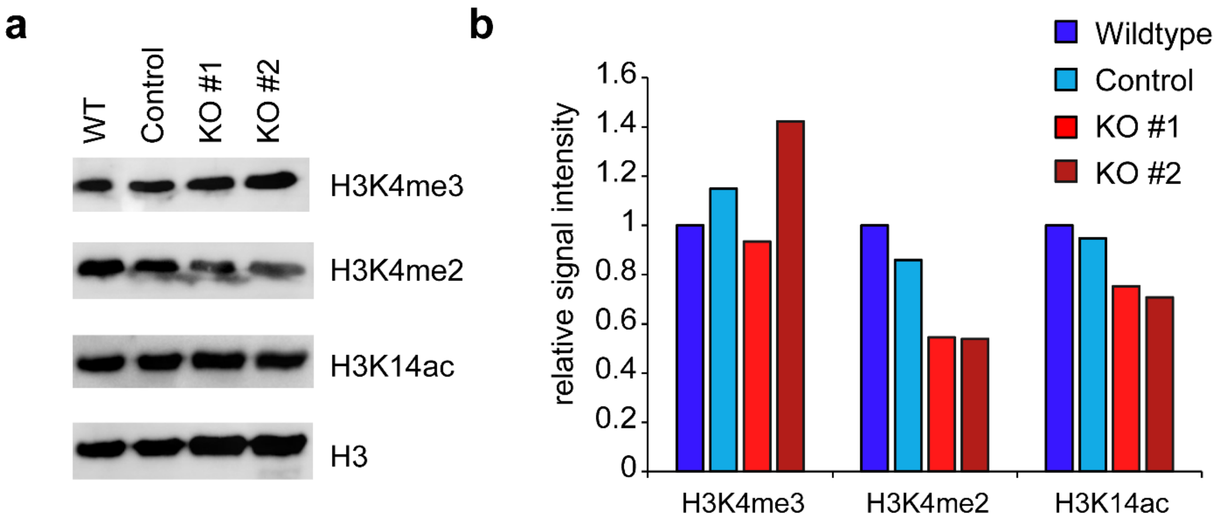

Figure S3. Global levels of histone modifications in SAMD1 KO HepG2 cells. **a** Western blotting of histone modifications in wildtype, control and SAMD1 KO cells. **b** Relative signal intensity, normalized to H3. .

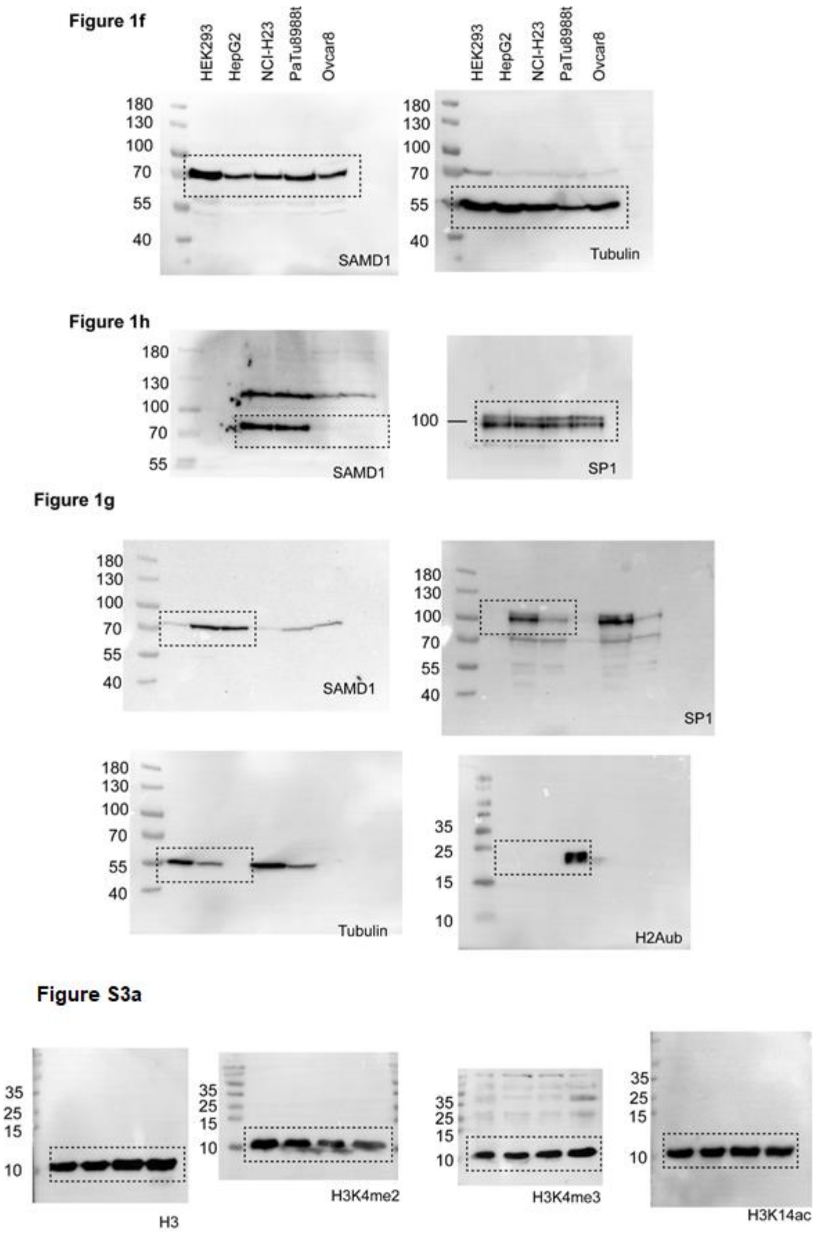

Figure S4. Uncropped original images.

Table S1. qPCR Primers.

**ChIP pPCR Primers**	
ChIP hNANOS1 fw	GCTCTGGTCTGCAGCCAATG
ChIP hNANOS1 rev	TTATCGGGCCCACTCCTCCC
ChIP hCBLN1 fw	GCGCCGGCTGCATCAATAAT
ChIP hCBLN1 rev	TCGCACTCCGGGACTAGCGT
ChIP Enhancer1 f	AAAGCAAGCAAAGACCCCAT
ChIP Enhancer1 r	CACTCCGCCCTTTTACTGTT
ChIP Enhancer2 f	GCGCGTTCCTCCTTTTACTC
ChIP Enhancer2 r	CCCTACCCCAAGACACTTCC
ChIP Enhancer3 f	TTTCCAAATTTACCACAGCGAA
ChIP Enhancer3 r	ACTATTGTGTTGCCTTTCAGAGT
ChIP Enhancer4 f	ACTGTTTGGAAGGACATGCC
ChIP Enhancer4 r	TGCTCACACTTCTTGCCTCT

ChIP Enhancer5 f	TCGCTTTGAAATCCTCCCCT
ChIP Enhancer5 r	TCAATGCCAGGGAAGAGGTT
**RT-qPCR Primers**	
RT hGAPDH f	AGCCACATCGCTCAGACAC
RT hGAPDH r	GCCCAATACGACCAAATCC
RT hB2M f	AGTATGCCTGCCGTGTGAAC
RT hB2M r	GGAGCAACCTGCTCAGATAC
RT hPIK3IP1 f	GCTACTCCTACAAGAGGGGG
RT hPIK3IP1 r	GGTCAACTGGAGTCTGGCTG
